# Insights into Nuclear Mitochondrial Sequence Distribution in the Pig Genome Based on the Latest Reference Assembly

**DOI:** 10.3390/ani16060919

**Published:** 2026-03-14

**Authors:** Hongtao Li, Cheng Yang, Guiming Zhu, Qin Zhang, Chao Ning, Dan Wang

**Affiliations:** College of Animal Science & Technology, Shandong Agricultural University, Taian 271000, China; 2023110343@sdau.edu.cn (H.L.); 2020010090@sdau.edu.cn (C.Y.); zhuguiming1929@163.com (G.Z.); qzhang@sdau.edu.cn (Q.Z.)

**Keywords:** NUMTs, pigs, genome, mitochondrial DNA

## Abstract

Horizontal transfer of mitochondrial DNA into the nuclear genome generates nuclear mitochondrial sequences (NUMTs), which serve as molecular fossils reflecting long-term mitochondrial–nuclear interactions and genome evolution. However, the biological mechanisms governing NUMT integration, retention, and evolutionary fate remain incompletely understood in domesticated animals. Here, using the latest pig reference genome assembly (Sscrofa11.1), we present a comprehensive genome-wide characterization of NUMTs in pigs and provide new insights into their genomic distribution and evolutionary constraints. We identified 513 high-confidence NUMTs, of which 460 were chromosomally mapped, accounting for 0.0106% of the nuclear genome. Beyond increased detection, our analyses reveal that pig NUMTs exhibit non-random origins, preferentially integrate into genomic regions under weak selective constraint, and are frequently associated with repetitive elements, consistent with a DNA repair-mediated insertion mechanism. NUMTs predominantly occur as short, fragmented sequences and show signatures of long-term neutral evolution, while insertions disrupting coding sequences are strongly selected against. Synteny-based analyses further identified clustered NUMT regions and duplicated NUMTs, suggesting secondary genomic duplication events following initial integration. Comparative analysis with the earlier Sscrofa10.2 assembly demonstrates that improved genome quality substantially enhances NUMT detection, particularly in repetitive and GC-rich regions, clarifying previously ambiguous sequence-context associations. Together, this high-quality pig NUMT map provides a robust foundation for future functional, evolutionary, and population-level investigations and contributes to the conservation and utilization of pig genetic resources.

## Simple Summary

During evolution, fragments of mitochondrial DNA occasionally integrate into the nuclear genome, forming nuclear mitochondrial sequences (NUMTs). Pigs are among the earliest domesticated livestock species and display extensive breed diversity. This study used the latest pig genome assembly to systematically find and analyze the distribution and characteristics of the NUMTs in the pig genome. We identified a total of 513 high-quality NUMTs distributed across the chromosomes. Our analyses revealed that these NUMTs were not generated randomly; instead, they preferentially originated from specific regions of the mitochondrial genome, and their nuclear insertion sites were associated with particular repetitive sequences. By leveraging a more accurate genome assembly, this study detected substantially more NUMTs than earlier versions of the pig genome, providing a more comprehensive landscape of NUMT integration. These findings enhance our understanding of pig evolutionary history and offer a valuable foundation for the conservation and utilization of pig genetic resources.

## 1. Introduction

Nuclear DNA sequences of mitochondrial origin (NUMTs) represent mitochondrial DNA (mtDNA) sequences that have transferred into the cell nucleus and integrated onto chromosomes [1]. The formation of NUMTs is an indispensable mechanism in the evolution of eukaryotes and their genomes [2,3,4,5]. Although NUMTs share high homology with mitochondrial sequences, their evolution proceeds at a significantly slower rate than mtDNA due to the slower evolutionary pace of nuclear DNA (nDNA). NUMTs carry more ancestral mitochondrial genetic information, serving as “molecular fossils.” They link maternally inherited mtDNA with Mendelian-inherited nDNA polymorphisms, forming crucial genomic coevolution [6]. Their presence–absence variation (PAV) can reveal overlapping biogeographic distribution histories between populations or varieties. These advantages make NUMTs a powerful tool in species evolution research [7].

In 1994, Lopez et al. first discovered and named NUMTs in their mitochondrial studies of felids [8]. To date, NUMTs have been discovered in hundreds of species, including animals, plants, and fungi, indicating that NUMTs are widely present in the genomes of eukaryotes. The distribution of NUMTs within the genome exhibits certain characteristics. In the human genome, the frequency of transposons within flanking sequences of NUMTs is significantly higher than the genome-wide average, with comparable frequencies upstream and downstream. However, no single transposon class—SINE, LINE, or LTR—exhibits a distinct difference. There is also no significant difference in the frequency of tandem repeats in NUMT flanking sequences and in the genome. The AT oligomer content in the flanking regions of NUMTs is higher than at the genomic level. Fewer NUMTs are generated by mitochondrial sequence translocation from the D-loop region [1].

Research on NUMTs in the pig genome remains limited. Schiavo et al. [9] analyzed genome-wide NUMT sequences in the pig Sscrofa10.2 reference genome, detecting a total of 430 NUMTs resulting from 246 mitochondrial sequence insertion events. The total length of these NUMTs covered 0.0078% of the genome, with their distribution being proportional to chromosome length. The longest NUMT sequence was located on chromosome 2, reaching a length of 111 kb. Detection results for NUMTs may vary across different genome versions [10]. The reference genome Sscrofa10.2 contains errors in completeness, sequence redundancy, order of sequence clusters, and orientation, which may lead to erroneous detection of NUMTs [9,11]. Therefore, this study utilizes the latest version of the pig reference genome from the NCBI to detect NUMTs in the pig genome, analyze their distribution characteristics, and lay the foundation for the conservation and utilization of pig germplasm resources.

## 2. Materials and Methods

### 2.1. Pig Reference Genome Sequence

The latest pig reference genome assembly (Sscrofa11.1, GenBank assembly accession: GCA_000003025.6) was obtained from the Ensembl database and used for the identification of NUMTs in pigs.

### 2.2. Detection of NUMTs in the Pig Genome

#### 2.2.1. Obtain Common Mitochondrial Sequences Shared by *Sus scrofa*

Considering the independent domestication origins of pigs in Europe and Asia [12] and to ensure comparable base dosage of European and Asian pigs in the shared sequences, mitochondrial genome sequences from 14 European and 14 Asian pigs were retrieved from public databases (Table S1). Multiple sequence alignments were generated using MAFFT software (v7.487) [13], and the mitochondrial consensus sequence for modern Eurasian pigs was obtained using CONS software (https://www.ebi.ac.uk/jdispatcher/msa/emboss_cons, accessed on 16 December 2025) [14].

#### 2.2.2. Avoid the Marginal Effects of mtDNA

Because the mitochondrial genome is circular in structure, if it is broken off at the start position of the control region and directly used for homology alignment, the levels of corresponding NUMTs at both ends of the mitochondrial genome may be underestimated. The mitochondrial genome sequence was linearized by joining its 5′ and 3′ ends to generate a linear mtDNA of double the original length. This linear mtDNA was then aligned against nDNA to identify NUMT sequences.

#### 2.2.3. Shielding Repetitive Sequences

When aligning two homologous sequences, simple repetitive sequences tend to overestimate similarity. Therefore, masking repetitive sequences or low-complexity sequences before performing homology alignment can improve the accuracy of NUMT detection. First, construct a repeat sequence library using RepeatModeler software (v2.0.4). Then, combine it with the RepeatMasker.lib library included with RepeatMasker software (v4.1.5) to filter out as many potentially interfering simple repeat sequences as possible.

#### 2.2.4. Homologous Alignment

Adjusting parameters for homology-based alignment can capture “ancient NUMTs” that exhibit lower similarity to modern mtDNA and were inserted into the nuclear genome at an earlier time. To facilitate distant homology detection, i.e., to identify early-arising NUMTs, the scoring system for base substitutions and gaps was revised such that matches were assigned +1, mismatches −1, gaps −7, and gap extensions −1. The NUMT sequences were detected in the pig genome through distant source comparison and their size, number, and density were quantified.

#### 2.2.5. Detection of False Positives

To assess the risk of false positive alignments, a decoy test method was employed. Since completely inverted mitochondrial genome sequences cannot arise through evolution, alignments obtained by comparing inverted mitochondrial sequences with the nuclear genome should yield false positives [9]. This method was used to detect false positives within NUMTs, utilizing the strictest E-value threshold to ensure a low false positive rate.

### 2.3. Analysis of the Distribution Characteristics of NUMTs

#### 2.3.1. Distribution of NUMTs on Chromosomes

We annotated the physical locations and lengths of NUMTs within the nuclear genome and calculated the number and total length of NUMTs on each chromosome. The proportion of each chromosome occupied by NUMTs was calculated by normalizing the total NUMT length to chromosome size. To assess whether NUMT distribution was associated with chromosome length, Pearson’s correlation analyses were performed between chromosome size and (i) the total length of NUMTs and (ii) the number of NUMTs per chromosome. Distribution plots and correlation analyses were conducted using R software (v4.4.0).

#### 2.3.2. Mitochondrial “Hotspots” for NUMT Insertion

All NUMTs were mapped onto the mitochondrial reference genome using MAFFT software. For each NUMT, the physical location and length within the mitochondrial genome were annotated. We statistically analyzed the frequency of NUMTs occurring within key mitochondrial regions, including the D-loop, the 13 polypeptide-coding genes, the 2 rRNA-coding genes, and the 22 tRNA-coding genes. Specifically, our statistical analyses encompassed NUMT length distribution, repetitive coverage proportions across the mitochondrial genome, coverage differences among genes of varying functional types, and identification of genes with the highest coverage. These analyses were used to identify potential “hotspot” regions for NUMT occurrence within the mitochondrial genome. In addition, the sequence characteristics of these hotspot regions were examined to explore features associated with elevated NUMT formation.

To quantify NUMT coverage over mitochondrial genes, homology mapping information for each NUMT was normalized by converting reverse-strand alignments to forward-strand orientation. Overlapping NUMT fragments were merged to avoid duplicate counting. Subsequently, a custom Python script (Python 3.10) calculated the proportion of genes covered by NUMTs on chromosomes and the proportion of genes covered by NUMT homologous sequences within mitochondria.

#### 2.3.3. Chromosomal Environment Preference

Based on the repetitive element annotations and positional information provided by the UCSC Genome Browser, we calculated the frequency of repetitive elements within 1000 bp flanking regions on both sides of each NUMT. In addition, the GC content of the 1000 bp upstream and downstream sequences of NUMTs was quantified. These metrics were used to examine the association between NUMT occurrence and specific sequence features and to statistically infer the sequence environment preferences of NUMTs.

### 2.4. NUMT Collinearity Merging

Mitochondrial DNA segments that insert into nuclear chromosomes may subsequently undergo structural variation along with the host chromosome. In this case, LAST (Local Alignment Search Tool), a local alignment software (v2.32.1) that employs an adaptive seed algorithm for sensitive homology search across entire genomes [15], will resolve a single fragmented NUMT insertion event into multiple discrete homologous matching sequences. Therefore, collinear sequence merging was performed based on the physical location of NUMTs in the reference genome to obtain the NUMT region.

Sequences of adjacent NUMTs with physical positions of less than or equal to 30 kb on the nuclear genome and homologous alignment to physical positions of less than or equal to 2.5 kb on the mitochondrial genome are classified as an NUMT region, which is considered to be the only insertion event that has undergone nuclear DNA insertion or deletion mutations. Among them, NUMTs with physical positions less than or equal to 2.5 kb on the mitochondrial genome are considered to be caused by nDNA deletion mutations; NUMTs with physical positions less than or equal to 30 kb on the nuclear genome are considered to be caused by nDNA insertion mutations.

In addition, there may be complex NUMT regions that have undergone structural mutations such as chromosome duplication and rearrangement due to one or more insertion events, i.e., NUMTs with physical positions of less than or equal to 30 kb on the nuclear genome but much greater than 2.5 kb on the mitochondrial genome.

### 2.5. NUMT Source Prediction

For each NUMT region, the sequences 200 bp upstream and downstream were extracted and concatenated. Pairwise sequence similarity was then assessed among all concatenated sequences. If two sequences exhibited both a similarity greater than 0.8 and coverage greater than 0.8, the corresponding NUMTs were classified as duplicated NUMTs, indicating that they likely underwent duplication events following chromosomal integration. Otherwise, they were considered unique or non-duplicated NUMTs, representing original insertions derived directly from mitochondrial fragments.

## 3. Result

### 3.1. NUMTs in the Pig Genome

**Figure 1:**
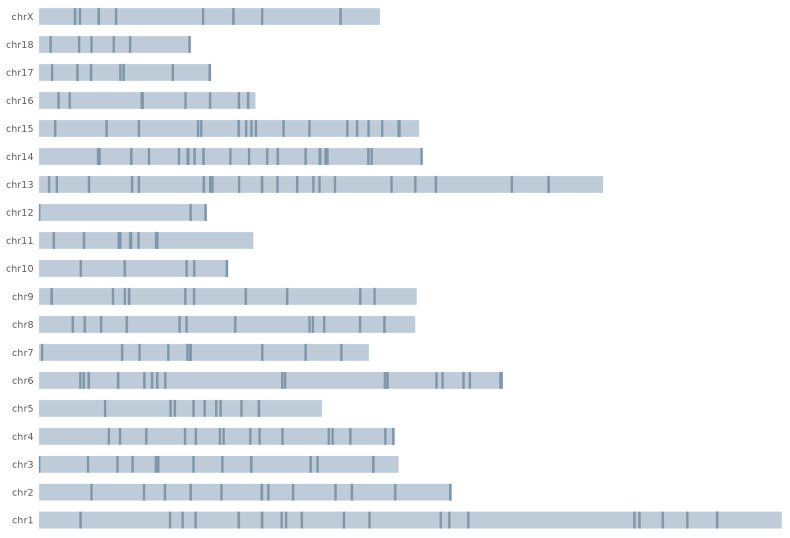
Distribution of NUMTs in the pig nuclear genome. NUMT positions are represented in the Sscrofa11.1 assembled chromosomes.

**Figure 2:**
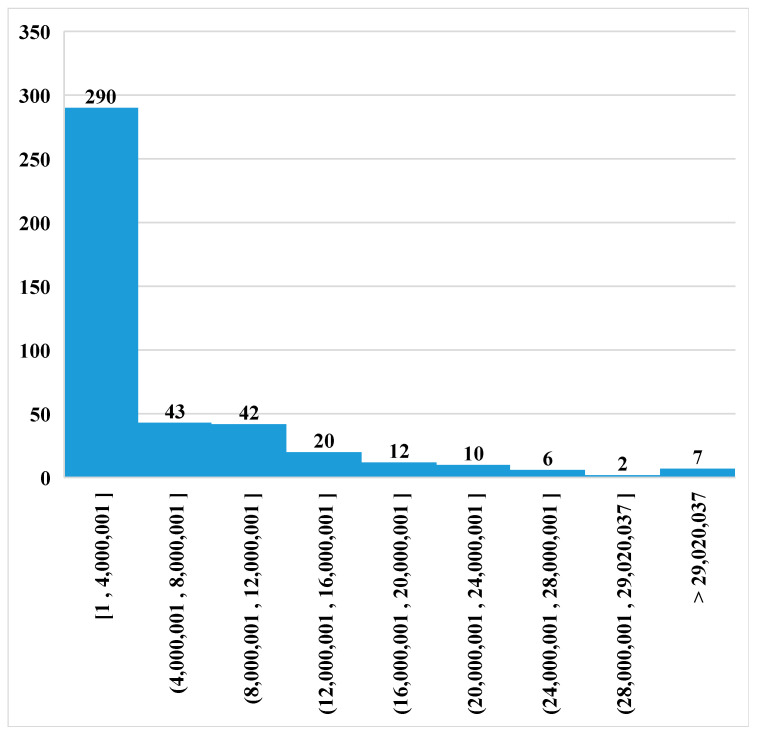
Distance between NUMTs.

The mitochondrial consensus sequence of modern Eurasian pigs was homologously aligned with the Sscrofa11.1 nuclear reference genome (613 sequences in total). A total of 530 NUMT sequences were identified with significant homology, of which 477 were located on chromosomes and 53 on scaffolds (Table S2). No alignments were detected when using the reverse mitochondrial sequence, indicating that no false-positive NUMTs were obtained. To ensure stringent quality control, NUMTs with an E-value > 1 × 10^−4^ were removed, resulting in the exclusion of 17 sequences. Ultimately, 513 high-quality NUMTs (LAST score > 61) were retained, including 460 located on chromosomes and 53 on scaffolds. Based on the total length of the Sscrofa11.1 nuclear genome (2,501,895,775 bp), NUMTs accounted for 0.0106% of the genome (=266,298 bp/2,501,895,775 bp). Figure 1 illustrates their chromosomal distribution. NUMT sequences showed occasional overlap on chromosomes, mostly involving only a few base pairs. The largest overlapping region spanned 52 bp and occurred on the X chromosome. The distances between adjacent NUMTs were generally small, with more than 90% of intervals shorter than 16 Mb. The maximum observed distance between tandem NUMTs reached 61 Mb (Figure 2).

The distribution of NUMTs across pig autosomes exhibits significant unevenness, with certain chromosomes (such as chromosome 14) carrying markedly higher numbers and total lengths of NUMTs, while others (such as chromosomes 16 and 18) harbor relatively fewer. A considerable number of NUMTs were also detected on the sex chromosome X (Table S3). There was a moderate strong correlation between the total length of NUMTs on each chromosome and the length of the chromosome (Pearson’s r = 0.40, *p*-value = 0.086) and a significant strong correlation between the number of NUMTs on each chromosome and their relative length to the chromosome (Pearson’s r = 0.66, *p*-value = 0.002).

The similarity between NUMTs and the mitochondrial consensus sequence of modern Eurasian pigs ranges from 62.28% to 100%, with an average similarity of 76.82%. NUMTs have a wide distribution in length, ranging from 39 to 11,182 bp, with an average length of 519 bp; the vast majority of NUMT sequences are 39 to 4800 bp long, and only one ultra-long NUMT sequence (11,182 bp) appears on chromosome 2, with a similarity of 90.75% to the modern consensus sequence (Table 1).

### 3.2. Genomic Environment Preferences of NUMTs

The GC content of the flanking sequences of NUMTs was calculated to determine whether there was any difference between the GC content of the entire genome. The results showed that the GC content of the upstream 1000 bp region of NUMTs was 0.4148, and the GC content of the downstream 1000 bp region was 0.4125, which was not much different from the GC content of the genome of 0.4187.

RepeatMasker was used to test whether there was any difference between the frequency of various repetitive elements flanking sequences of NUMTs and the proportion of repetitive elements in the entire genome. The results showed that the repetitive sequence content of the upstream 1000 bp region of NUMTs was 0.4445, and the GC content of the downstream 1000 bp region was 0.4374, which was not much different from the genomic GC content of 0.4404. Counting the number of occurrences of each repeat element, it was found that the three common types of retrotransposons-short interspersed nuclear elements (SINEs), long interspersed nuclear elements (LINEs) and long terminal repeats (LTRs), and simple repeats (Simple_repeat) appeared most frequently on the flank of NUMTs, with 473, 279, 157, and 217, respectively, among which SINE/tRNA, LINE/L1, and Simple_repeat had the most (Table 2).

### 3.3. NUMT Region

**Figure 3:**
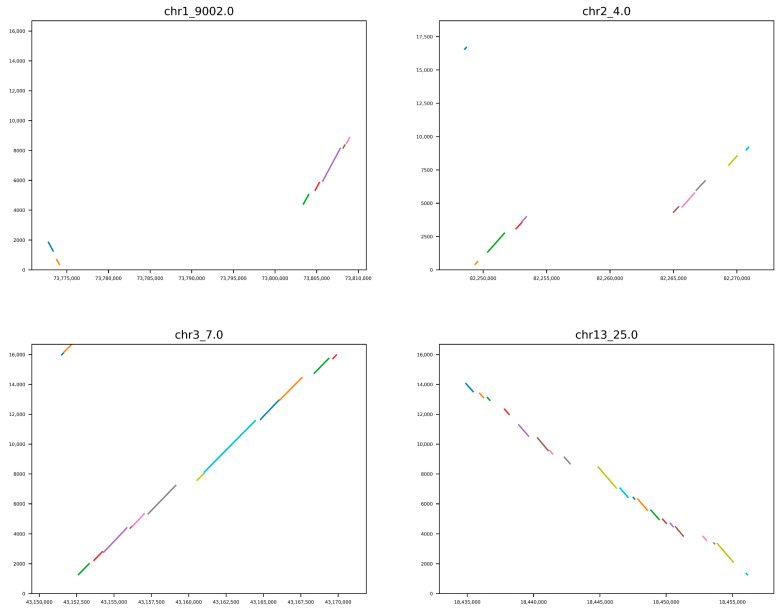
A few NUMT regions. NUMT region ID and numt sequences are reported at the top of each plot. The *x* axis reports the positions in the indicated chromosomes. The *y* axis reports the mtDNA sequence coordinates. Different colored lines in the plots indicate different NUMT sequences. In each panel, the slope of the line reflects the orientation of the NUMT insertion. A positive slope indicates an NUMT region in the same orientation as the mitochondrial reference, while a negative slope (e.g., the NUMT region on chr13) indicates an inverted orientation.

Collinear merging of NUMT sequences yielded 240 NUMT insertion events, i.e., 69 NUMT regions that integrated at least two NUMT sequences (61 NUMT regions were found on chromosomes, 8 on scaffolds, and some NUMT regions are shown in Figure 3), and 171 NUMTs were singleton NUMTs. Among them, 48 NUMT areas are formed by a single insertion event, and 21 are complex insertion events. Most NUMT regions (88.52%) contain fewer than nine NUMT sequences. The number of NUMT regions on each chromosome was significantly and strongly correlated with the length of NUMTs (Pearson’s r = 0.87, *p*-value = 1.72 × 10^−6^) and with its relative length (Pearson’s r = 0.82, *p*-value = 1.77 × 10^−5^). The average distance between adjacent NUMT areas is 28.12 Mb.

### 3.4. NUMT Sources

We found that the similarity between five pairs of NUMTs was above 0.8 (considering that the similarity between NUMTs and the mitochondrial consensus sequence of modern Eurasian pigs was at least 62.28%, and the maximum reached 100%, the average similarity was 76.82%), and the coverage was above 0.8 (Table S4).

### 3.5. Mitochondrial DNA Regions of NUMT Origin

**Figure 4:**
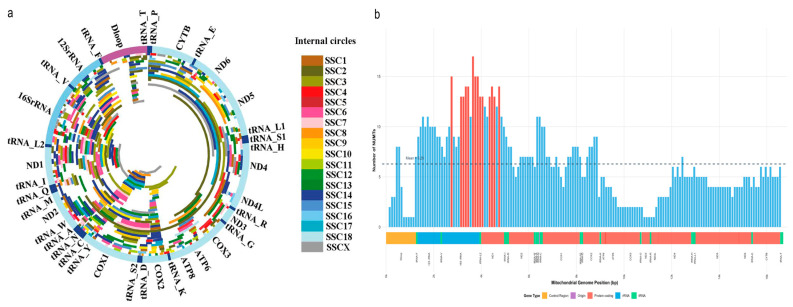
Distribution and insertion frequency of NUMTs in the pig mitochondrial genome. (**a**) Circular map of the pig mitochondrial genome. NUMTs are color-coded according to the pig chromosome into which they are inserted. Mitochondrial genes are displayed in the outer ring region. (**b**) Quantitative distribution of NUMTs along the mitochondrial genome. The *x* axis represents mitochondrial DNA positions. Each bar corresponds to a 100 bp window, with height indicating the number of NUMTs within that window. Red regions denote high-count windows, and dashed lines mark the global mean (Mean = 6.29).

According to the distribution of the mitochondrial homologous sequences of NUMTs in the mitochondrial genome, it can be seen that almost the entire mitochondrial genome is covered by NUMT sequences at least once (Figure 4). The mtDNA regions most covered by NUMTs include parts of the *16SrRNA*, *COX1*, *ND1*, *COX2*, and *ND2* genes, covered more than 50 times.

To further analyze the evolutionary constraints on NUMTs after integration into the nuclear genome, we examined their structural features and overlap with functional regions. Structural comparisons of three representative NUMTs (NUMT314, NUMT359, and NUMT445, located at chr14:21808471-21808667, chr14:70688062-70688137, and chrX:15135619-15135851, respectively) located in different nuclear genes revealed that all were fully contained within non-coding regions (introns or UTRs). Compared to their mitochondrial homologs, they retained high structural integrity and sequence similarity (86.7–97.97%) and did not overlap with any exons.

Therefore, we instead compared the distribution proportions of NUMT sequences across two functional genomic regions. Notably, 99.42% of NUMT homologous sequences originated from mitochondrial gene regions, whereas only 30.38% of NUMTs within the nuclear genome were located inside gene regions.

### 3.6. Comparison with Sscrofa10.2 Genomic NUMTs

**Figure 5:**
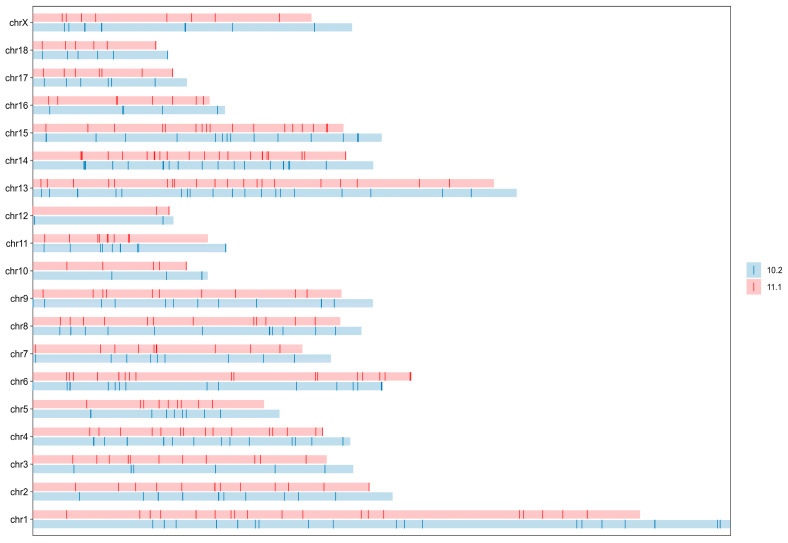
Distribution of NUMTs in Sscrofa10.2 and Sscrofa11.1.

Comparing the NUMT sequences identified in the reference genomes of Sscrofa11.1 and Sscrofa10.2, it was found that 513 and 435 NUMT sequences were detected in Sscrofa11.1 and Sscrofa10.2, respectively. The total length of NUMTs is 266,298 bp and 216,323 bp, respectively. Moreover, 69 and 61 NUMT regions containing more than one NUMT sequence were identified, respectively. It can be seen from the distribution map of NUMTs on chromosomes (Figure 5) that the difference in the distribution of NUMTs on the two sets of chromosomes mainly occurs at the ends of chromosomes and other positions.

## 4. Discussion

Nuclear mitochondrial sequences (NUMTs) arise from the transfer of mitochondrial DNA fragments into the nuclear genome, a process widely attributed to DNA double-strand break repair, primarily via non-homologous end joining [16]. In this framework, free mitochondrial DNA released during cellular stress or mitochondrial turnover can be opportunistically integrated into the nuclear genome, a process facilitated by local genomic instability and repetitive or structurally complex regions.

Consistent with this mechanism, the genome-wide NUMT map generated in this study reveals widespread and non-random mitochondrial-to-nuclear DNA transfer in the pig genome. Nearly the entire mitochondrial genome is represented within NUMTs, and the unequal contribution of different mitochondrial regions supports previous observations that NUMT formation frequencies vary among mitochondrial loci [7]. Unlike reports in some primate genomes [1], we did not observe substantial NUMT underestimation associated with the highly mutable D-loop region, suggesting that NUMT detection in pigs is not strongly biased by mitochondrial mutation rate.

Following integration, NUMTs exhibit distinct evolutionary fates depending on their genomic context. The majority of NUMTs inserted into intergenic or non-coding regions experience weak selective constraints and evolve largely neutrally [10], undergoing progressive fragmentation and sequence decay. This is consistent with the predominance of short NUMT fragments and their enrichment in non-coding regions observed in this study. In contrast, NUMT insertions that disrupt coding sequences are strongly selected against, explaining their near absence from exons, while a small subset retained within introns or untranslated regions may persist and, in rare cases, acquire regulatory or structural roles [17,18]. In repeat-rich regions, secondary duplication events may further amplify certain NUMTs, contributing to local copy number variation [19,20].

Comparative analyses across domestic animals indicate that pigs harbor a relatively high number of NUMTs compared with several other species [21,22,23,24,25]. Differences in NUMT abundance appear to be partially associated with genome size and chromosome length [7,9,26], consistent with previous studies [10], although variation among species with similar genome sizes suggests that additional factors, such as transposon activity and genome turnover rates, also influence NUMT accumulation [27].

Importantly, the use of the latest Sscrofa11.1 reference genome substantially increased NUMT detection compared with earlier analyses based on Sscrofa10.2 [28], highlighting the critical role of reference genome quality in accurately characterizing NUMT landscapes, particularly in repetitive and GC-rich regions [9,11].

Taken together, these observations support a unified conceptual model in which NUMTs originate through DNA repair-mediated integration, are preferentially retained in genomic regions under weak selective constraint, and are progressively shaped by neutral evolution and local duplication events. While evolutionary and domestication-related implications of NUMTs have been proposed in previous studies [9,26,29,30,31], the present work does not directly test such hypotheses. Interpretations based on NUMT distribution patterns—such as potential signals of interspecific admixture [9] or domestication-related differentiation [30]—should therefore be regarded as literature-informed extrapolations rather than conclusions supported by direct evidence. Accordingly, the primary contribution of this study lies in providing a high-quality, genome-wide NUMT reference map for pigs, together with a conceptual framework for interpretation. Further validation of the proposed evolutionary implications will require population-scale analyses, functional genomic data, and cross-species comparisons in future studies.

## 5. Conclusions

This study identified genome-wide NUMTs in pigs using the Sscrofa11.1 assembly and characterized their distribution. NUMT length correlated with chromosome length, with hotspots found in retrotransposon-rich regions but not associated with GC content. Structural variation fragmented or duplicated NUMTs after nuclear insertion, and their mitochondrial origins were non-random. These findings provide insights into NUMT evolution and contribute to understanding pig genetic diversity and germplasm conservation.

## Figures and Tables

**Table 1:** Statistical information on the length of NUMTs and their similarity to mitochondrial homologous sequences.

Number	Size Range (bp)	Similarity Range (%)
66	39–99	75–100
150	100–249	64.62–100
131	250–499	63.24–94.14
68	500–749	62.28–99.81
29	750–999	63.89–100
58	1000–1999	64.02–99.86
10	2000–4999	67.09–91.06
1	>5000	90.75

**Table 2:** Statistics of the number of repeated elements in upstream and downstream flanking sequences of NUMTs.

Repeat Elements	Number Occurred
DNA/hAT-Blackjack	2
DNA/hAT-Charlie	27
DNA/hAT-Tip100	7
DNA/PiggyBac	1
DNA/TcMar-Tc1	1
DNA/TcMar-Tc2	2
DNA/TcMar-Tigger	7
LINE/CR1	4
LINE/L1	227
LINE/L2	43
LINE/RTE-BovB	2
LINE/RTE-X	3
Low_complexity	24
LTR/ERV1	87
LTR/ERVK	21
LTR/ERVL	25
LTR/ERVL-MaLR	24
None	70
Satellite	2
Simple_repeat	217
SINE/MIR	59
SINE/tRNA	414
snRNA	3
tRNA	47

## Data Availability

All sequence data analyzed in this study are publicly available. The pig reference genome assembly (Sscrofa11.1) was obtained from the Ensembl database. Mitochondrial genome sequences were retrieved from the National Center for Biotechnology Information (NCBI). The specific accession numbers used in this study are provided in Supplementary Table S1.

## Supplementary Materials

The following supporting information can be downloaded at: https://www.mdpi.com/article/10.3390/ani16060919/s1, Table S1: Mitochondrial genome information for the construction of a consensus sequence across Eurasian pigs; Table S2: List of identified NUMTs in the pig genome; Table S3: Distribution characteristics of NUMTs in chromosomes; Table S4: Information of duplicated NUMTs.
